# Continuing Challenges in Rural Health in the United States

**Published:** 2019-12-16

**Authors:** Steven S. Coughlin, Catherine Clary, J. Aaron Johnson, Adam Berman, Vahe Heboyan, Teal Benevides, Justin Moore, Varghese George

**Affiliations:** 1 Department of Population Health Sciences, Medical College of Georgia, Augusta University, Augusta, GA; 2 Institute of Public and Preventive Health, Augusta University, Augusta, GA; 3 Division of Cardiology, Medical College of Georgia, Augusta University, Augusta, GA; 4 College of Allied Health Sciences, Augusta University, Augusta, GA; 5 Department of Occupational Therapy, College of Allied Health Sciences, Augusta University, Augusta, GA

## Introduction

Estimates of the total U.S. population living in non-metropolitan (rural) counties vary from 46.2 million to 59 million people. This represents 14% to 19% of the U.S. population. A recent AAMC report (Warshaw, 2017) addresses some of the challenges of rural health and associated health disparities affecting millions in the U.S. Rural populations are culturally heterogeneous, are spread broadly across large areas of the U.S., and have different demographics (Douthit et al., 2015). Compared to urban areas, rural communities face higher poverty rates, lower educational attainment, lack of transportation, a higher proportion of elderly individuals, and lack of access to health services (Hunsaker & Kantayya, 2010; Ricketts, 2000). Owing to these factors, rural communities face elevated rates of morbidity and mortality and greater percentages of excess deaths from the five leading causes of death including cancer and cardiovascular disease (Garcia et al., 2019).

Rural older adults have a higher prevalence of several chronic diseases compared to urban older adults, including coronary heart disease and diabetes (Kumar & Acanfora, 2001; O’Connor & Wellenius, 2012). Diabetes, one of the leading causes of death in the U.S., has been reported to be as much as 17% higher in rural areas than in urban areas. Compared to urban areas, reductions in diabetes mortality are lagging in rural areas (Callaghan et al., 2019). Diabetes was ranked as the third most important rural priority in Rural Healthy People 2020 (Bolin et al., 2015). Rural older adults are also more likely to be obese, the prevalence of which ranged from 24% in urban counties to 29% in rural counties (Cohen et al., 2017).

Several studies have found that rural cancer patients are more likely to be diagnosed at a later stage (Lin & Wimberly 2017; Sankaranarayanan et al., 2009). However, other studies found a reverse pattern that urban areas had a higher risk of late- stage diagnosis compared with rural areas (McLafferty et al., 2009; McLafferty et al., 2011). Rural residents may have long travel times to reach oncology care and they may have other health care access barriers such as low income, low education, poor health literacy, or disability (Fairfield et al., 2019).

Rural patients have an estimated 8% to 15% increased risk of death from colon cancer (Andrilla et al., 2019). In rural areas, lung cancer mortality is up to 20% higher than in metropolitan areas (Atkins et al., 2017). Several factors contribute to the high burden of lung cancer in rural areas, including disproportionately higher smoking rates (Doogan et al., 2017). In addition, residents of rural areas may have less access to public health interventions and health care services aimed at lung cancer prevention, screening and treatment (Fairfield et al., 2019). Differential access to evidence-based care may also increase lung cancer mortality in rural areas. Timeliness of medical care for newly diagnosed cancer is important for optimizing clinical outcomes (Fairfield et al., 2019). Treatment delays for lung cancer are partly due to system-level factors such as the care setting, existing referral resources, and the availability and quality of diagnostic and treatment services (Olsson et al., 2009).

In addition to health care for chronic diseases (heart disease, diabetes, cancer), rural residents also face challenges in receiving care for mental health conditions as well as autism and neurodevelopmental disabilities. Autism affects 1 in every 59 children in the U.S., according to estimates from the Centers for Disease Control and Prevention’s Autism and Developmental Disabilities Monitoring Network in 2018 (Ning et al., 2019; McBain et al., 2019). Although similar rates of autism are reported in rural and urban areas, rural families report greater difficulty in accessing resources. An overwhelming number of families experience long waitlists for diagnostic and therapeutic services (Ning et al., 2019). Among children with autism, caregivers report that access problems are exacerbated by services not being available in a given area, and these issues are greater for those children on the spectrum than those with other special health care needs (Benevides et al., 2016). Among adults on the autism spectrum, access to care is a significant problem; however, little is known about the contribution of rural or urban status on access for adults with autism and other neurodevelopmental conditions. Some authors posit that geographical differences are an area for future work to understand access problems among adults on the autism spectrum (Taylor & Henninger, 2015).

Access to mental health services in rural areas is a significant issue. Mental Health and mental disorders was ranked as the fourth most important rural priority in Rural Healthy People 2020 (Bolin et al., 2015). With few programs in rural areas, rural residents are further limited by a lack of transportation or resources to obtain transportation to reach the services that are available (Priester et al., 2016). This issue is particularly evident among youth where rural suicide rates are nearly double the urban rate for both males and females and longitudinal analyses suggest that the disparity is increasing (Fontanella et al., 2015).

Rural areas have relatively high levels of morbidity and mortality accompanied by the fewest health care resources (Ricketts, 2000). The distribution of physicians and other health care providers has remained skewed towards urban areas (Gong et al., 2019). Residents of rural areas often have more restricted access to health insurance than their urban counterparts (Nganje & Addey, 2019). Rural health clinics and federally qualified health centers play an important role in addressing rural health care.

The rural health care system in the U.S. has changed dramatically over the past two decades because of a transformation of health care financing, the introduction of new technologies, the clustering of health services into systems and networks, and the passage of the Affordable Care Act, which extended health insurance to previously uninsured/underinsured individuals (Ricketts, 2000; Callaghan, 2019). Rural health care changed as a result of the increased integration of health care professionals and institutions into systems and networks (Moscovice et al., 1997). Rural health professionals have chosen to join into systems and alliances to cope with the turbulent environment of health care policy and economics (Ricketts, 2000). By combining resources, rural providers expect to reduce their costs, manage their scarce resources, and compete effectively (Ricketts, 2000).

Government policies have attempted to address rural health disparities by encouraging network development and telemedicine, and by changing the rules for Medicare payments to providers (Ricketts, 2000). Telemedicine continues to grow in importance in rural health, as providers and patients become more familiar with systems and technologies and grow more comfortable with the capabilities of telecommunications (Ricketts, 2000).

To address pressing rural health concerns in the U.S., continued policy development and policy-oriented health services research is needed. This includes research on disparities in chronic diseases (heart disease, diabetes, cancer), as well as neurodevelopmental disorders, with the goal of assisting providers and decision/policy-makers at the federal, state and local levels to better understand problems faced by rural communities and to provide information that will be applied in ways that improve both health care access and population health.
